# The challenge of antioxidants to free radicals in periodontitis

**DOI:** 10.4103/0972-124X.44100

**Published:** 2008

**Authors:** Gowri Pendyala, Biju Thomas, Suchetha Kumari

**Affiliations:** *Department of Periodontics, A. B. Shetty Memorial Institute of Dental Sciences, Derlakatte, Mangalore, Karnataka, India*; 1 *Department of biochemistry, K. S. Hegde Medical Academy, Derlakatte, Mangalore, Karnataka, India*

**Keywords:** Antioxidants, free radicals, periodontal disease, oxidative stress

## Abstract

Periodontal disease is a chronic adult condition. Bacteria implicated in the etiology of this disease causes destruction of connective tissue and bone. As a result of stimulation by bacterial antigen PMN produces free radicals via respiratory burst as a part of host response to infection. Patients with periodontal disease display increased PMN number and activity. This proliferation results in high degree of free radical release culminating in heightened oxidative damage to gingival tissues, periodontal ligament and alveolar bone. Damage mediated by free radicals can be mitigated by “ANTIOXIDANT DEFENSE SYSTEM “. Physiological alteration and pathological states produced by free radicals depend on disequilibrium between free radical production and antioxidant levels leading to oxidative stress.

Hence this study has been designed to estimate the TOTAL ANTIOXIDANT CAPACITY in patients with PERIODONTITIS and healthy control subjects

## INTRODUCTION

Oxygen is required for all living organisms for their survival. But at the same time it is potentially toxic. Salvemini has described oxygen as a double edged sword. It is vital to life but at the same time because of its highly reactive nature it is capable of becoming part of potentially damaging molecules called free radicals. Oxygen is the ultimate electron acceptor in mitochondrial electron transport chain where flow of electrons ultimately produces energy in the form of ATP. The leaked out electrons are exposed to oxygen leading to formation of free radicals.

**Figure 1:**
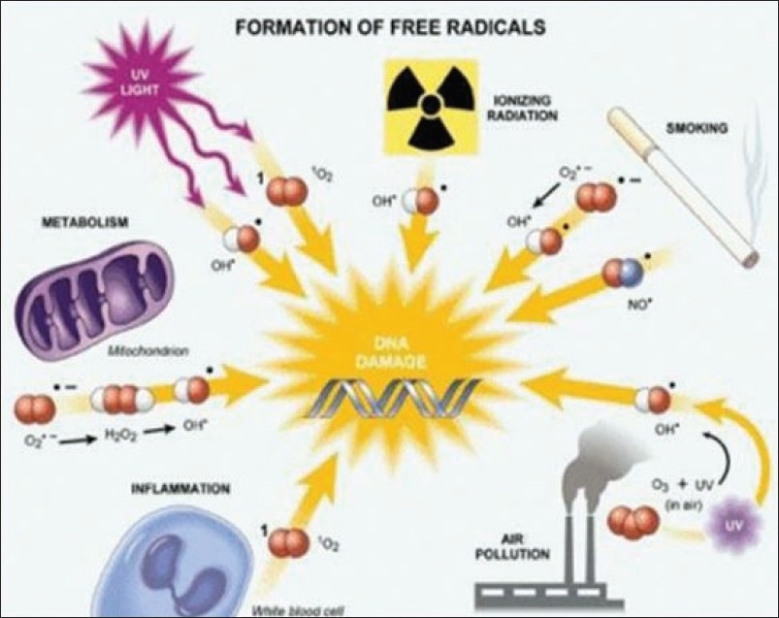
Formation of free radicals

A free radical may be defined as “any species capable of independent existence that contains one or more unpaired electrons”.[1] This makes it extremely reactive towards other molecules. Living cells are exposed to oxidants originating from a large variety of exogenous or endogenous sources [Figure 1].

Exogenous sources - air pollutants, ozone, radiation, chemicals, toxins, pathogenic microorganisms.[2]

### Endogenous sources[2]

- due to leaks in electron transport chain in mitochondria during oxidation of food stuffs
- inflammatory cells produce free radicals by a process of respiratory burst during phagocytosis
- enzymes which indirectly produce free radicals.

Free radicals cause tissue damage by a variety of different mechanisms which include

- DNA damage
- lipid peroxidation
- protein damage
- oxidation of important enzymes [e g anti proteases]
- stimulation of pro inflammatory cytokines release

Reactive oxygen species [ROS] encompasses other reactive species which are not true radicals but are nevertheless capable of radical formation in the intra and extra cellular environment. E. g: Hydrogen peroxide, Hypochlorous acid, singlet oxygen, ozone.[2]

The living organism has adapted itself to an existence under a continuous efflux of free radicals. Among the different adaptive mechanism the antioxidant defense mechanism is of major importance.

Antioxidants are “those substances which when present in lower concentrations compared to those of an oxidisable substrate, will significantly delay or inhibit oxidation of that substrate”.[1]

The different possible mechanisms by which antioxidants may offer protection against free radical damage include[3]

- prevention of formation of free radicals
- interception of free radicals by scavenging the reactive metabolites and converting them to less reactive molecules
- facilitating the repair of damage caused by free radicals
- providing a favourable environment for effective functioning of other antioxidants.

Antioxidant defense system is very dynamic and responsive to any disturbance taking place in redox balance of body. Antioxidants can be up regulated and neutralize free radicals formation that could take place due to oxidative stress. Transcription factors such as nuclear factor -kb and activating protein 1 are redox sensitive.[2] Redox potential is a measure of the affinity of a substance for electrons.

Smaller changes in redox state - trigger gene transcription events which lead to tissue damage secondary to induction of pro inflammatory state.[2]

**Figure 2:**
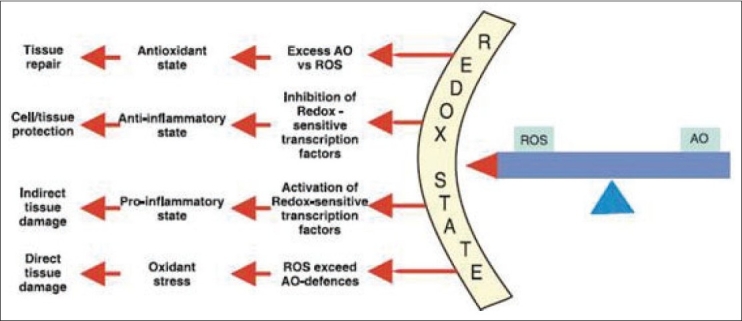
The biologic effects of small and large shifts in the balance between free radicals and antioxidants

Larger upward shift in the pro oxidant / antioxidant ratio bring direct damage to vital biomolecules and structures [Figure 2].[2]

**Figure 3:**
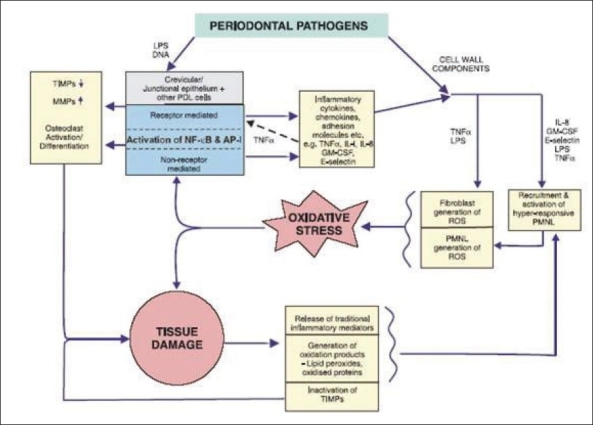
Oxidative stress and tissue damage

The body has a sophisticated antioxidant defense system to cope with free radical formation under normal conditions and thereby maintaining redox balance. However, when there is not an excess of antioxidant defense and an overproduction of free radicals or a drop in level of antioxidants it will lead to an imbalance and cause deleterious effects a situation known as oxidative stress [Figure 3].[4]

Periodontitis is a term used to describe an inflammatory process initiated by plaque biofilm that leads to loss of periodontal attachment to root surfaces and adjacent alveolar bone which ultimately results in loss of tooth.[2]

The primary etiological agent is specific, predominantly gram negative anaerobic facultative bacteria within subgingival biofilm.[5] These bacteria have the ability to activate host defense mechanisms which breakdown epithelia and other structures of gingiva and periodontium, while at the same time inactivating repair systems. Bacteria cause tissue destruction directly by toxic products and indirectly by activating host defense mechanisms.[6]

Among the host responses leukocytes serve as the initial host defense against periodontal pathogens. After stimulation by bacterial pathogens neutrophils produce free radicals. Periodontal tissue destruction is caused by an inappropriate host response to these microorganisms and their products. More specifically due to oxidative stress.

Hence this study has been designed to estimate the serum total antioxidant capacity in periodontitis and health.

### Objectives of the study

The objectives of the study is to investigate

1. Total antioxidant capacity in serum of patients with periodontal disease.
2. Total antioxidant capacity in serum of patients without periodontal disease.
3. To compare the total antioxidant capacity in serum of patients with and without periodontal disease.

## MATERIALS AND METHODS

### Source of data

Subjects reporting to Department of Periodontics , A.B. Shetty Memorial Institute Of Dental Sciences , Mangalore

### Method of collection of data

Sample size of 60 subjects are taken and divided into 2 groups of 30 each

Group 1: Control group: 30 subjects with healthy periodontal conditions

Group 2: Study group: 30 subjects with clinically diagnosed periodontitis

## CRITERIA FOR SELECTION

### Inclusion criteria

1. Clinical attachment loss ≥5mm measured using Williams periodontal probe
2. Bleeding on probing
3. Controls who are periodontally healthy
4. Patients who had not undergone any periodontal treatment for atleast 6 months prior to sampling
5. All measurements and samples are taken before starting any periodontal therapy

### Exclusion criteria

1. Subjects who require antibiotic or anti inflammatory drug therapy
2. History of any systemic disease
3. Subjects who are pregnant and pre eclamptic
4. Subjects with a history of smoking and tobacco consumption
5. Subjects with vitamin supplements
6. Subjects who regularly use mouth washes.

### Investigations

The total antioxidant capacity of clinical samples is measured using spectrophotometric quantitation through formation of phosphomolybdenum complex.

Venous blood samples collected are centrifuged at 3000 rpm for 15 minutes and the supernatant serum is collected.

An aliquot of 0.1.ml of sample solution containing a reducing species (in water, methanol, ethanol, dimethylsulfoxide) was combined in an eppendrof tube with 1ml of reagent solution (0.6M sulfuric acid, 28mM sodium phosphate, and 4mM ammonium molybdate).

The tubes are capped and incubated in a thermal block at 95 degree centigrade for 90 minutes. After the samples are cooled to room temperature, the absorbance of aqueous solution of each was measured at 695nm against a blank.

## RESULT

A study was conducted in Department of Periodontics, A. B. Shetty Memorial Institute of Dental Sciences, Mangalore to evaluate and compare Total Antioxidant Capacity in serum in chronic periodontitis patients and healthy subjects with a sample size of 60 each subdivided into case and control groups of 30 each.

The group statistics showed a mean of 28.5052 for cases and 50.5955 for controls in serum [Table 1]. The student ‘t’ test [Table 2] was used for statistical analysis with 95% of confidence interval showed significant difference between total antioxidant capacity in serum of case and control groups, with p value .000 at baseline in terms of age ,number of teeth, oral hygiene status, systemic conditions, smoking and pan chewing.

**Table 1:** Group statistics

	Group	N	Mean	Std. deviation	Std. error mean
Anti-oxidant μg/dl	Case	30	28.5052	4.6283	0.8594
	Control	30	50.5955	3.1371	0.5825

**Table 2:** Independent samples test

	t-test for equality of means			
	
	t	*P*-value	Mean difference	95% confidence interval of the difference	
				
				Lower	Upper
Anti-oxidant μg/dl	-21.276	.000	-22.0903	-24.1702	-20.0104

## DISCUSSION

The present study was conducted in the department of Periodontics, A.B. Shetty Memorial Institute Of Dental Sciences, Mangalore to evaluate the total antioxidant capacity in serum in chronic periodontitis patients and healthy subjects with a sample size of 60 subdivided into case and control of 30 each showed statistically significant difference between case and control groups.

Reactive oxygen species are associated with pathogenesis of variety of inflammatory diseases and have a role (direct or indirect) in tissue damage.

Periodontal disease occurs in predisposed individuals with an aberrant inflammatory and immune response to microbial plaque.[2] Neutrophils are the predominant inflammatory cells in gingival tissues.[7]

Chronic inflammatory conditions are associated with increased oxidative stress with phagocytes [particularly neutrophils] being implicated in disease pathogenesis because of generation of oxidative burst during phagocytosis and killing.[2]

Plaque bacteria and their products are source of factors that could stimulate neutrophils infiltrating the periodontal tissues.[2] Enhanced free radical generation by neutrophils can be stimulated with bacteria associated with periodontal disease.[8]

Diseased sited will be associated with increased levels of a variety of cytokines and chemokines produced by inflammatory cells and normal resident cell population with in periodontal tissues.[2]

A variety of pro inflammatory cytokines [TNF ALPLA, IL-8, IL-1, IL-6], growth factors and lipopolysaccharides have a priming effect on human neutrophil oxidative burst.[9]

Although all cells produce ROS during normal physiological functions[10] it is phagocytes that produce high levels to facilitate the killing and destruction of microbes.[11]

Majority of tissue destruction in periodontitis is considered to be the result of an aberrant inflammatory / immune response to microbial plaque and involve prolonged release of ROS and neutrophil enzymes.[2]

ROS generation in periodontal disease causes bone resorption, degrade connective tissue, increases matrix metallo proteinases activity causing an imbalance.[2]

Traditionally ROS production by phagocytes has been associated with the defense of body to infection as they are essential for efficient killing of microbes.[2]

By contrast, ROS generation at high levels can cause oxidative stress with in tissues and result in direct damage to cells and extracellular matrix.[2] Products of this oxidative damage such as advanced glycation end products and lipid peroxide proteins can lead to further ROS induced damage by their priming and chemotactic effect on neutrophils.[2]

Nuclear factor kb and activator protein, the two redox sensitive transcription factors are of potential importance in the pathogenesis of periodontal disease.[2]

They can be activated by a variety of stimuli including bacterial products, viral proteins, cytokines, growth factors, oxidative stress.[12] After activation they regulate transcription of genes important in inflammation, tissue remodeling and repair.[2]

**Graph 1:**
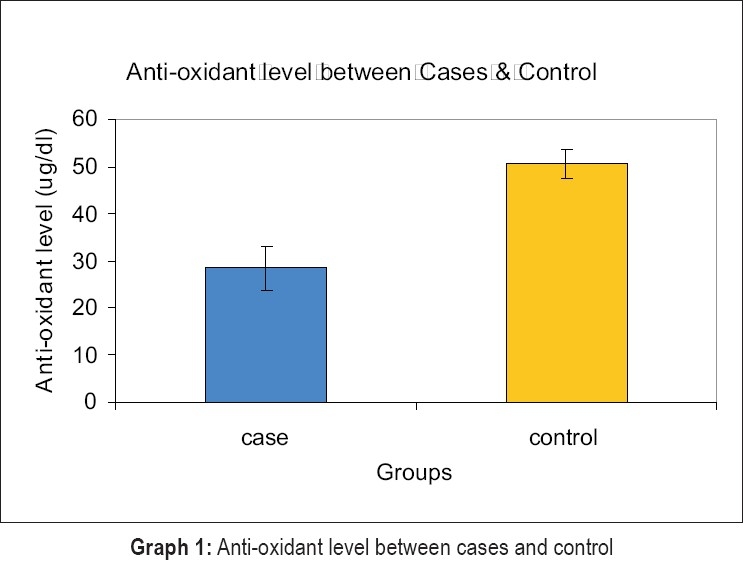
Anti-oxidant level between cases and control

In our study the results showed that the total antioxidant capacity in serum in periodontitis patients was significantly lower when compared to healthy subjects [Graph 1]. The results are consistent and in support with the following trials.

Broke *et al*. [2004] studied the total antioxidant capacity of serum in periodontitis and control subjects and found higher serum total antioxidant capacity for healthy controls than periodontitis cases.[3]

Pavlica *et al*. [2004] investigated total antioxidant capacity of serum and concluded that the total antioxidant capacity in periodontitis was lower than in health and suggested a negative correlation between total antioxidant capacity and periodontal parameters.[13]

Chapple *et al*. [1997] studied serum samples in periodontitis and control groups and concluded that prevalence of periodontitis was positively associated with decreased serum antioxidant capacity.[14]

## CONCLUSION

Oxidative stress lies at the heart of periodontal tissue damage that results from host microbial interactions, either

- as a direct result of excessive ROS activity
- antioxidant deficiency
- activation of transcription factors and the creation of pro inflammatory state

while a myriad of possible mechanisms leading to periodontal destruction exist, the influence of free radicals and antioxidants cannot be overlooked undoubtedly.

Several avenues of enquiry now exist for the development of antioxidant based approaches to periodontal therapy which includes traditional routes of increasing the antioxidant capacity of periodontal tissues and newer routes based on modulation of transcription factors

This array of pathways provides opportunities to develop novel antioxidant therapies that target the free radicals and which function not only as antioxidants in the traditional sense but also as powerful anti inflammatory agents.
